# Spinel LiMn_2_O_4_ as a Capacitive Deionization Electrode Material with High Desalination Capacity: Experiment and Simulation

**DOI:** 10.3390/ijerph20010517

**Published:** 2022-12-28

**Authors:** Yuxin Jiang, Ken Li, Sikpaam Issaka Alhassan, Yiyun Cao, Haoyu Deng, Shan Tan, Haiying Wang, Chongjian Tang, Liyuan Chai

**Affiliations:** 1 School of Metallurgy and Environment, Central South University, Changsha 410083, China; 2 College of Engineering, Chemical and Environmental Engineering Department, University of Arizona, Tucson, AZ 85721, USA; 3 Chinese National Engineering Research Center for Control and Treatment of Heavy Metal Pollution, Changsha 410083, China; 4 Water Pollution Control Technology Key Lab of Hunan Province, Changsha 410083, China

**Keywords:** capacitive deionization, LiMn_2_O_4_, lithium ion, desalination capacity, simulation

## Abstract

Capacitive deionization (CDI) is a newly developed desalination technology with low energy consumption and environmental friendliness. The surface area restricts the desalination capacities of traditional carbon-based CDI electrodes while battery materials emerge as CDI electrodes with high performances due to the larger electrochemical capacities, but suffer limited production of materials. LiMn_2_O_4_ is a massively-produced lithium-ion battery material with a stable spinel structure and a high theoretical specific capacity of 148 mAh·g^−1^, revealing a promising candidate for CDI electrode. Herein, we employed spinel LiMn_2_O_4_ as the cathode and activated carbon as the anode in the CDI cell with an anion exchange membrane to limit the movement of cations, thus, the lithium ions released from LiMn_2_O_4_ would attract the chloride ions and trigger the desalination process of the other side of the membrane. An ultrahigh deionization capacity of 159.49 mg·g^−1^ was obtained at 1.0 V with an initial salinity of 20 mM. The desalination capacity of the CDI cell at 1.0 V with 10 mM initial NaCl concentration was 91.04 mg·g^−1^, higher than that of the system with only carbon electrodes with and without the ion exchange membrane (39.88 mg·g^−1^ and 7.84 mg·g^−1^, respectively). In addition, the desalination results and mechanisms were further verified with the simulation of COMSOL Multiphysics.

## 1. Introduction

Water demand is rising quickly due to increased industrial production and urbanization, thus culminating in a severe shortage of fresh water for people to use [1,2]. Various methods have been developed for desalination to alleviate the crisis, such as membrane distillation, multi-effect distillation, multi-stage distillation, reverse osmosis (RO), etc. [3]. These methods are operated under high temperature or high pressure, resulting in high energy consumption and high maintenance costs [4,5]. On the other hand, capacitive deionization (CDI) is a novel desalination technology with low energy consumption, low cost, high efficiency, good regeneration ability, and mild working conditions [6,7]. A CDI system usually consists of two electrodes at different sides of the feed solution. When the current goes through, the salt ions are adsorbed by the electrodes with the energy being stored in the system. The ions and energy are released when the opposite current passes, thus regenerating the electrodes conveniently without secondary pollution. The working voltage of the system is usually about 1.0 V, which is energy-efficient and avoids the splitting of water.

The electrode materials are essential to CDI performances. Traditional CDI electrodes are carbon-based materials [8], which have good cycling abilities but relatively low desalination capacities [9,10]. The salt adsorption capacity of carbonaceous material is usually under 15 mg·g^−1^ [11,12], limiting the application of CDI. The desalinating processes of CDI are comparable to the charging processes of supercapacitors and batteries from the perspective of energy transmission, so the CDI electrode materials and battery materials may be closely related, offering researchers a variety of opportunities for improving CDI performances. The battery materials have larger electrochemical capacities than carbon-based materials because of the larger scale of charge transfer generated by redox reactions, indicating better desalination capacities [13]. Sodium-ion battery materials have been attractive in the CDI research due to their big family and the intercalation and deintercalation behaviors of sodium ions. NaMnO_2_ [14] was employed as an electrode in a hybrid capacitive deionization (HCDI) system (a system with a battery electrode and a capacitor electrode) and reached a high desalination capacity of 31.2 mg·g^−1^. The aqueous sodium-ion battery anode NaTi_2_(PO_4_)_3_ [15] could be used as a CDI electrode with activated carbon (AC) as the counter electrode, and an excellent salt removal capacity of 140 mg·g^−1^ was exhibited. Chloride-ion battery materials have also drawn more and more attention owing to their high desalination performances. Chang constructed a CDI system with commercially purchased bismuth [16] as the electrode for the capture of chloride ions and showed a good desalination capacity of 55.52 mg·g^−1^, which was about three times that of AC.

Higher CDI performances are possible if the ion transfer is improved. We notice that most of the battery materials in CDI belong to sodium- or chloride-ion battery materials [17], thus sodium or chloride ions have been the mostly used charge carriers in CDI. Compared to a sodium or chloride ion, a lithium ion seemed a better charge carrier which has the same amount of charge but a much lower weight [18]. A better charge carrier could lead to more efficient ion transfer. There are mainly four types of lithium-ion battery (LIB) materials in industrial production right now: lithium iron phosphate (LiFePO_4_) [19], lithium cobalt oxide (LiCoO_2_) [20], spinel lithium manganese oxide (LiMn_2_O_4_) [21], and ternary cathode material [22]. Among these materials, LiMn_2_O_4_ is a cathode material with the advantages of low cost, good stability in aqueous media, and a long voltage plateau inside the voltage window of water, which makes it an ideal CDI electrode material candidate [23,24]. Compared to lithium manganese oxide (LMO) materials of other morphologies, the spinel LMO has not only a high theoretical electrochemical capacity of 148 mAh·g^−1^ but also a stable 3-dimensional structure which allows the facile transportation of lithium ions, further facilitating the ion transfer in the CDI system.

In this work, we figured out a routine of using the spinel LMO in the CDI system with an anion exchange membrane to enhance the desalination performance. Unlike many other CDI electrode materials desalinating saline solution through direct intercalation or adsorption of salt ions, spinel LMO removed the negative ions of the other side of the anion exchange membrane with the charge balance effect in the solution by releasing positive ions into its own side, revealing a new way of utilizing LIB materials in desalination techniques. The process of deionization was simulated by the software COMSOL Multiphysics and an excellent desalination capacity of 159.49 mg·g^−1^ was obtained in the experiment.

## 2. Materials and Methods

### 2.1. Materials

Sodium chloride (NaCl, analytical reagent, Sinopharm, Beijing, China) was used to prepare the feed solutions in CDI experiments. Lithium chloride (LiCl, ≥99%, Aladdin, Shanghai, China) was used to prepare the electrolytes in electrochemical characterization experiments and the solutions in the CDI experiments. Lithium manganese oxide (LiMn_2_O_4_, spinel, Ziyi, Shanghai, China) was employed as the cathode material in the CDI system without further purification. AC (XFP01, XFNANO, Nanjing, China) was employed as the anode material in the CDI system. Anion exchange membrane (AMX, ASTOM, Tokyo, Japan) was used to separate LiCl solution and NaCl solution in the desalination process. N-methylpyrrolidone (NMP, analytical reagent, Aladdin, Shanghai, China), conductive carbon black (TIMCAL Graphite & Carbon, Bodio, Switzerland), and polyvinylidene fluoride (PVDF, HSV900, MTI Corp., Richmond, USA) were used for the preparation of the electrodes.

### 2.2. Characterization

The structure and morphology information of materials was obtained with a field-emission scanning electron microscope (FESEM, MIRA3 LMH, TESCAN, Brno-Kohoutovice, Czech) and X-ray diffraction (XRD, TTR-3, Rigaku, Tokyo, Japan), and the masses of the XRD samples of spinel LMO before and after desalination were controlled to be equal. X-ray photoelectron spectroscopy (XPS) measurements were operated on an X-ray photoelectron spectrometer (Thermo Scientific K-alpha, Thermofisher, Waltham, USA) with Mono AlKα as the X-ray source to get the valence information of the spinel LiMn_2_O_4_. The tap densities of the materials were tested by a tap density tester (BT-302, Bettersize, Dandong, China). The surface area and pore size analyzer (KUBOX1000, Bjbuilder, Beijing, China) was employed to measure the specific surface area (SSA) and pore size distribution (PSD) with the Brunauer–Emmett–Teller (BET) method. The PSD information was further analyzed with a Barret–Joyner–Halenda (BJH) method.

### 2.3. Preparation of Electrodes

The active material, PVDF, and carbon black were mixed with a mass ratio of 8:1:1 and ground in an agate mortar for about 10 min. The mixture was then dissolved in NMP and stirred for 5 min to form a homogeneous slurry, which was further painted onto the surface of current collectors and put into a vacuum oven at 90 °C for 4 h to totally remove the residual solvent, forming the final electrode. As for the electrode in the electrochemical test, the slurry was painted onto carbon paper (TORAY, H-060, Japan). Besides, the slurry was coated onto the square of 5.0 cm × 5.0 cm on the titanium plate for the CDI experiment. The total mass of the electrode materials in each desalination test was controlled to be about 100 mg, and the mass ratio of cathode and anode active materials was about 1:2.

### 2.4. Electrochemical Tests

All the electrochemical tests were carried out with an electrochemical workstation (Multi autolab/M204, Metrohm, Herisau, Switzerland). Cyclic voltammetry (CV) and galvanostatic charge–discharge (GCD) tests were conducted to study the redox behaviors and electrochemical capacities of the materials. These experiments were conducted in a 3-electrode system with the active material as the working electrode, a platinum plate as the counter electrode, a silver/silver chloride electrode (Ag/AgCl, in a saturated KCl solution) as the reference electrode, and 1.0 M LiCl aqueous solution as the electrolyte. The scan rate was 0.5 mV·s^−1^ during the CV experiment. The current density in the GCD experiments was 100 mA·g^−1^ with the voltage range of 0~1.0 V. The specific capacities of the materials were calculated based on GCD results with the following equation:

(1) $$C_{s}=\left(I\times \Delta t\right)/m,$$

in which $C_{s}$ (mAh·g^−1^) refers to the specific capacity of the active material, I (A) denotes the current applied to the material, Δt (h) is the discharging time, and m (g) represents the mass of the active material.

### 2.5. Capacitive Deionization Experiments

**Figure 1:**
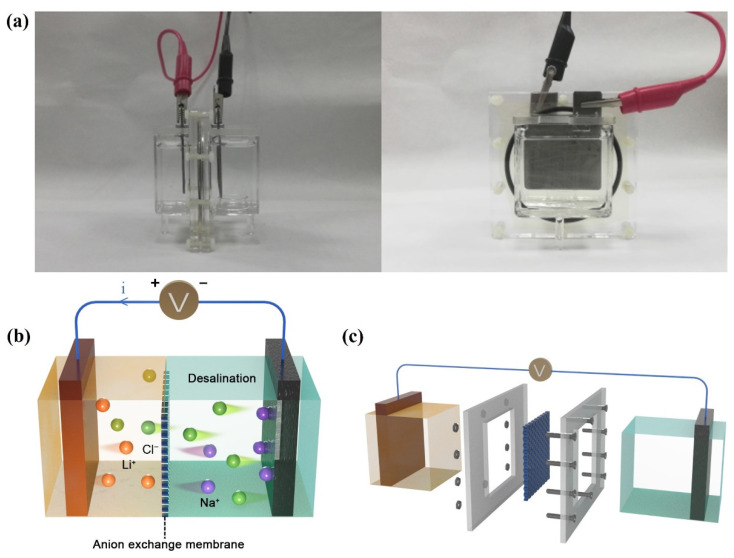
(**a**) Photographs of the CDI device; (**b**) the schematic illustration of the desalination process; and (**c**) the assembling of the desalination system.

As presented in Figure 1a, CDI desalination experiments were conducted in a membrane capacitive deionization cell with a batch mode. The whole cell was divided into two compartments by the anion exchange membrane, one with 100 mL 10 mM LiCl aqueous solution and the other with 125 mL NaCl aqueous solution as the feed solution to be desalinated. The membrane was fixed inside the glass container with a rubber band and the container was assembled with non-conductive plastic screws. The spinel LMO material was employed as the cathode and immersed in the LiCl solution, while the AC was immersed in the NaCl solution and used as the anode. Figure 1b displays how the NaCl solution was desalinated. When a positive voltage was charged onto the cathode, lithium ions were released from spinel LMO to attract the chloride ions on the other side to go through the anion exchange membrane, and the sodium ions were adsorbed onto the AC anode, thus desalinating the NaCl solution. The membrane blocked the lithium and sodium ions from penetrating into the opposite compartment, providing a driving force for the movement of chloride ions. The salt was released back into the desalinated solution when an opposite current went through. Interestingly, it was a liquid region instead of a material surface that adsorbed the chloride ions during desalination, which could break the limitations of surface adsorption.

The CDI system was charged with a constant voltage by the electrochemical workstation while the ion concentration changes in the NaCl solution and the LiCl solution were analyzed by ion chromatography (883 Basic IC Plus, Metrohm, Herisau, Switzerland). The pH values of the solutions were tested with a pH tester (PHSJ-3F, Leici, Shanghai, China). The desalination capacity of the system was calculated as follows:

(2) $$SAC=\left(C_{0}-C\right)\times V/m,$$

where SAC (mg·g^−1^) denotes the salt removal capacity, $C_{0}$ (mg·L^−1^) and C (mg·L^−1^) refer to the initial and final concentrations of NaCl solution in the CDI test, V (L) is the volume of the NaCl solution, and m (g) demonstrates the total mass of the active materials of the CDI cell. Additionally, the salt adsorption rate was obtained with the equation:

(3) SAR=SAC/t

in which SAR (mg·g^−1^·min^−1^) represents the salt adsorption rate and t (min) denotes the operation time of the CDI reaction.

### 2.6. Simulation Method

**Figure 2:**
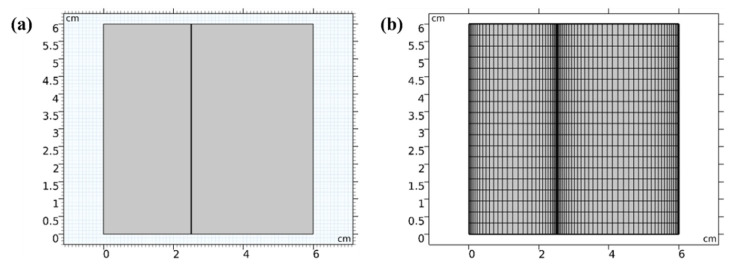
(**a**) Geometry of the CDI device; (**b**) meshes in the device defined in the geometry section.

A COMSOL Multiphysics (COMSOL, Inc., Stockholm, Sweden) simulation was employed to examine the ion behaviors in the desalination processes and further support the deionization experimental findings. Due to the horizontal direction of the electrical field in the CDI cell, we employed a 2-dimensional model to simulate the reaction in the device. The model’s structure is depicted in Figure 2a; the device had a square shape with a side length of 6.0 cm, with the LiCl solution compartment (2.5 cm × 6.0 cm) on the left and the NaCl solution compartment (3.5 cm × 6.0 cm) on the right. While the right side of the NaCl solution was set to be the anode plate with AC at 0 V, the left side of the LiCl solution was set to be the cathode plate with LMO material at 1.0 V. The anion exchange membrane separating the components was 0.2 mm thick.

The preprocessing method of calculation known as “lattice subdivision” involved breaking up the entire calculation region into a number of smaller ones. We used reflection to separate the CDI device into rectangular structures, with the biggest unit size being 3.18 mm and the smallest being 0.018 mm (Figure 2b). The unit’s curvature factor was 0.7 and its maximum growth rate was 1.3. The precision of the calculations at the boundary was further improved by using arithmetic progression and symmetric distributions, which provided more available calculation points and units.

The calculation of the desalination device was based on the aqueous electric neutrality equation in the tertiary current distribution, including the transportation of H^+^ and OH^−^. The pH was 7 during the desalination process, which was verified by experiment. In order to depict the ion flux through the ion exchange membrane with the existence of ion concentration and electric field gradients, the Nernst–Planck Equation (Equation S2) was used in the model to calculate the transfer of the ions in the solutions. Furthermore, the boundary conditions are listed in Table S1.

## 3. Results and Discussion

### 3.1. Enhanced Ion Transfer by LIB Materials

To confirm the role of lithium ion in CDI desalination, exploratory CDI experiments were conducted with AC as both electrodes (at 1.0 V with the initial NaCl concentration of 10 mM, Figure S1). A low desalination capacity of 7.84 mg·g^−1^ was obtained without the incorporation of ion exchange membranes. In contrast, a higher desalination capacity of 26.59 mg·g^−1^ was achieved in the CDI cell with an anion exchange membrane, which was in accordance with a mechanism about ion exchange membranes in CDI as reported previously [25,26,27]. The anion exchange membrane could effectively save energy in CDI by blocking the cations from leaving the cathode side, and still allow the anions to penetrate into the LiCl solution, resulting in a better desalination capacity. Moreover, by replacing the NaCl solution in the cathodic region with 10 mM LiCl solution, the AC electrodes showed an even higher salt removal capacity of 39.88 mg·g^−1^, signifying that ion transfer in CDI could be improved with lithium ions as charge carriers.

**Figure 3:**
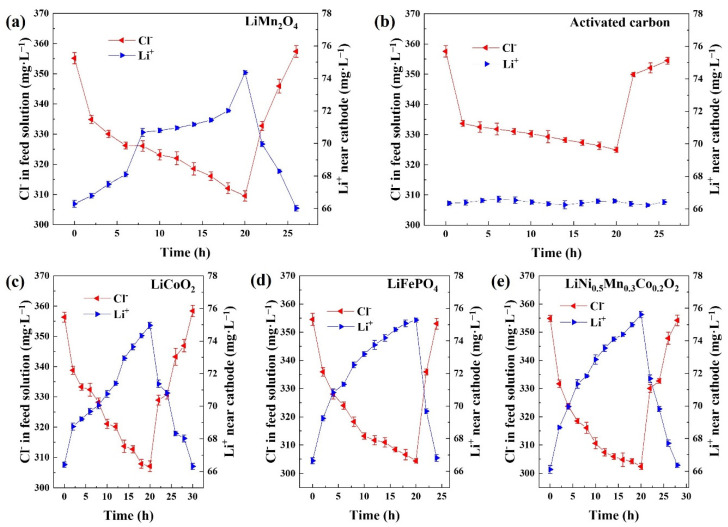
(**a**–**e**) The concentration variations of chloride ions in the feed solution and lithium ions in the LiCl solution during CDI desalination with different cathodes (voltage: 1.0 V, initial salt concentration: 10 mM).

Four typical LIB electrodes (LiMn_2_O_4_, LiCoO_2_, LiFePO_4_, and LiNi_0.5_Mn_0.3_Co_0.2_O_2_) and AC were employed as cathodes in the HCDI tests, respectively. The concentration variations of chloride ions in the feed solution and lithium ions in the LiCl solution are presented in Figure 3. All the CDI tests with LIB electrodes exhibited stronger transfer of chloride ions in comparison with the CDI test with AC cathode. For the experiments with battery electrodes, the concentrations of lithium ions rose during the desalination stage and dropped during the recovery stage. Simultaneously, the concentrations of chloride ions dropped during desalination and increased back to the pristine levels after the recovery processes. The release of lithium ions was beneficial for the transfer of chloride ions. The feasibility of improving the desalination performances by employing LIB electrodes was confirmed.

The variation ranges of the chloride ion concentrations in CDI tests with different LIB cathodes were similar. However, owing to the high cost of cobalt, LiCoO_2_ and LiNi_0.5_Mn_0.3_Co_0.2_O_2_ might not be ideal CDI electrode candidates. Moreover, the tap density of LiFePO_4_ is relatively low compared to other LIB cathodes, which is detrimental to the future development of delicate CDI devices. The tap density of commercial LiFePO_4_ is generally below 0.8 g·cm^−3^ [28]. In this study, the tap density of LiFePO_4_ was 0.8 g·cm^−3^, 57.89% lower than that of LiMn_2_O_4_ (1.9 g·cm^−3^). On the contrary, LiMn_2_O_4_ is a cheap, green cathode material with appropriate tap density and capacity, which was chosen for further research as a CDI electrode.

### 3.2. Structure and Morphology

**Figure 4:**
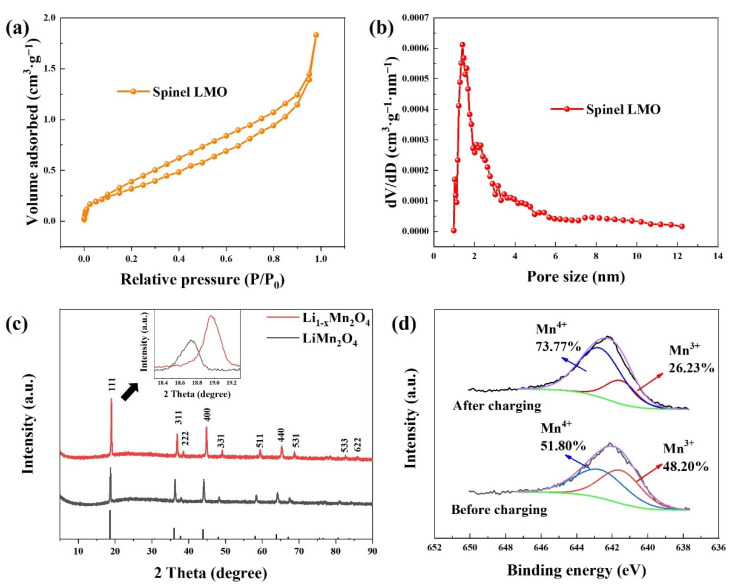
(**a**) N_2_ adsorption/desorption curve and (**b**) pore size distribution of the LiMn_2_O_4_ material; (**c**) the XRD patterns of the spinel LMO electrode and (**d**) the XPS profiles of Mn 2p_3/2_ before and after the deionization process.

Figure S2 illustrates the typical spinel morphology of the LiMn_2_O_4_ material under FESEM observation, which provides the material with good stability. In the crystal structure of the spinel LiMn_2_O_4_, manganese ions located just inside the octahedrons consist of oxygen ions. The vacancies between these ions offer channels for the fast diffusion of lithium ions. The sizes of the particles were at the level of μm as shown in Figure S2b,c. The pore sizes of LiMn_2_O_4_ were mainly below 10 nm (Figure 4b) and a typical IV isotherm was exhibited (Figure 4a) [29]. The SSA of the spinel LMO material was 0.87 m^2^·g^−1^ according to the BET result.

To achieve an initial understanding of the redox behaviors of the spinel LMO material during the desalination process, XRD and XPS tests were conducted to examine the material before and after the deionization process of applying 1.0 V for 20 h. The diffraction peaks moved rightwards after charging in Figure 4c, which meant that the interplanar distances of the material reduced, demonstrating the release of lithium ions. Besides, the intensity of the peaks rose after charging, signifying the exposure of the crystal faces of the spinel LMO. It was noticeable that the intensity of the (111) facet increased more than other facets, which suggested that the (111) lattice plane was an active facet that participated in lithium-ion extraction. In the XPS spectra, the peaks of Mn^3+^ and Mn^4+^ were at the binding energy of 641.5 eV and 642.7 eV, respectively [30]. As demonstrated in Figure 4d, the content of Mn^4+^ increased to 73.77% after the charging process, owing to the electrochemical oxidation of manganese ions. Nevertheless, the manganese in the LiMn_2_O_4_ was not oxidated to a full extent; Mn^3+^ still kept a 26.23% share of the total manganese, which also implied that an improvement could be still possible with the extension of desalination time.

### 3.3. Electrochemical Behaviors

**Figure 5:**
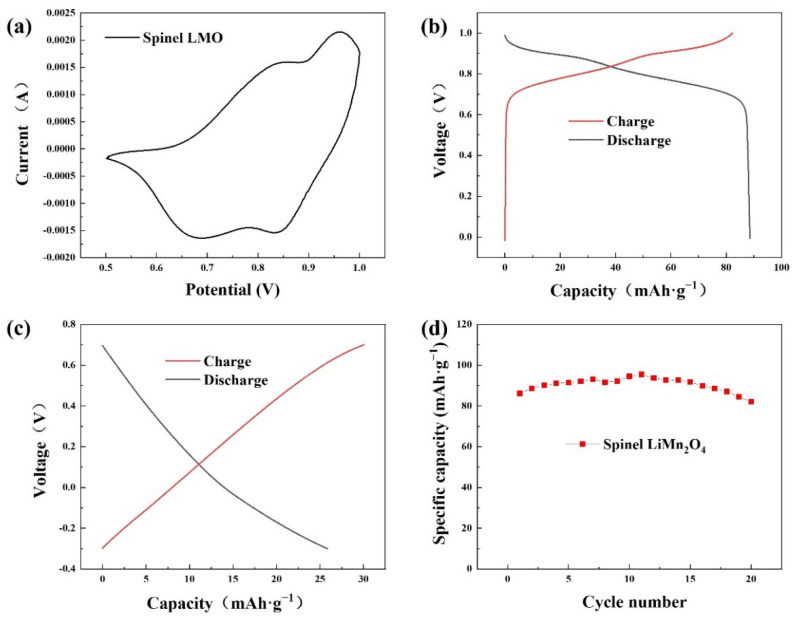
(**a**) CV curve of spinel LMO; GCD curves of (**b**) the spinel LMO material and (**c**) AC; (**d**) cycling performance of spinel LiMn_2_O_4_ over 20 cycles in 1.0 M LiCl solution.

The CV curve of spinel LiMn_2_O_4_ at 0.5–1.0 V is illustrated in Figure 5a with the upper voltage limited to 1.0 V to avoid water electrolysis. The CV profile exhibits two pairs of redox peaks. The pair at the higher voltage denoted the lithiation/delithiation processes occurred at the 8a site of the LMO crystal structure, while the other pair at the lower voltage revealed the corresponding processes at the 16c site. These peaks referred to the reversible redox behaviors of Mn^3+^/Mn^4+^ in LMO, in accordance with the processes of release and capture of lithium ions represented by Equations (4) and (5).

Lithium release:

(4) $$LiMn_{2}O_{4}\to Li_{1-x}Mn_{2}O_{4}+xLi^{+}+xe^{-}$$

Lithium capture:

(5) $$Li_{1-x}Mn_{2}O_{4}+xLi^{+}+xe^{-}\to LiMn_{2}O_{4}$$

The GCD profiles of the spinel LMO and AC in a 1.0 M LiCl aqueous solution are illustrated in Figure 5b,c. The charge and discharge curves of the spinel LMO are symmetrical, representing good reversibility. The voltage range of the electrochemical plateaus corresponding to the redox reactions of manganese ions was between 0.65 V and 1.0 V. On the other hand, the GCD profile of the AC electrode was composed of two straight lines, demonstrating that no redox reactions existed on the electrode, which was the common electrical double-layer behavior of carbon-based material. The capacity of spinel LiMn_2_O_4_ was 88.67 mAh·g^−1^, more than three times that of AC (25.86 mAh·g^−1^), which revealed the good potential of enhancing the desalination capacity by employing spinel LMO as a CDI electrode. The voltage ranges of the GCD tests were different for the two materials. A higher voltage might cause severe oxygen evolution for the common AC electrode because the oxygen evolution potential is about 0.7 V versus Normal Hydrogen Electrode (NHE) in a neutral solution [31], while 1.0 V is appropriate for LiMn_2_O_4_ due to the oxygen evolution overpotential [32]. The cycling ability was also tested by the GCD method as exhibited in Figure 5d. The capacity barely decreased after 20 cycles, indicating good electrochemical stability of LMO.

### 3.4. Desalination Performances

**Figure 6:**
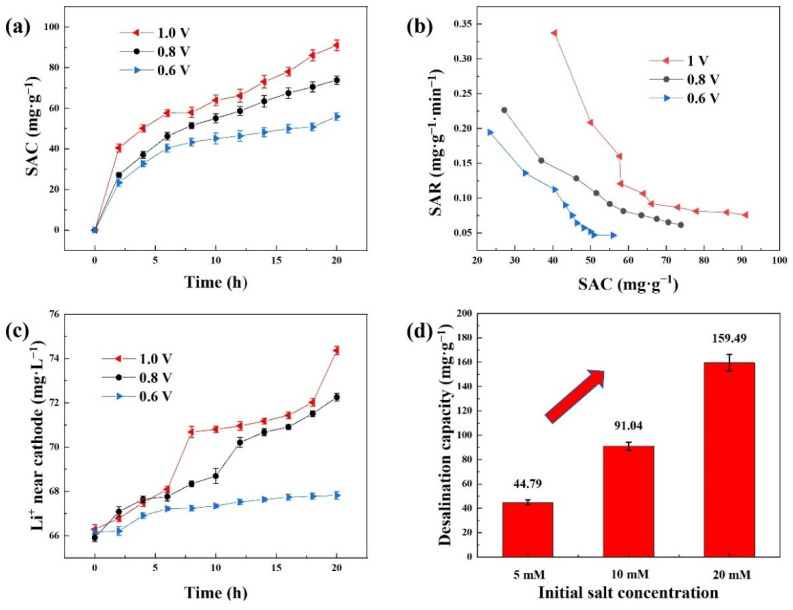
(**a**) Desalination capacities of the CDI cell over 20 h in 10 mM NaCl solution with the constant voltage mode (1.0 V, 0.8 V, 0.6 V); (**b**) SAR-SAC Ragone plots; (**c**) concentration changes of the lithium ions in 10 mM LiCl solution during the CDI processes; (**d**) desalination capacities of different initial NaCl concentrations at 1.0 V.

Figure 6a displays the SAC curves of the spinel LMO over 20 h with different applied voltages, and the initial concentrations of the salt solutions were 10 mM. A high desalination capacity of 91.04 mg·g^−1^ was obtained after 20 h at 1.0 V. As the operation time increased, the salt removal capacities became larger, and the tendency of the rising of the desalination capacities did not stop even though the desalination process had been conducted for 20 h. In addition, the higher the voltage was, the larger capacity the CDI system would perform with. It was obvious that a higher-applied voltage would provide a stronger electric field driving the salt ions. Moreover, it could be inferred from the GCD profile of spinel LiMn_2_O_4_ (Figure 5b) that more lithium ions could be released from the cathode with a higher voltage. Both could make more chloride ions penetrate through the anion exchange membrane, leading to a larger desalination capacity. The rate of salt removal decreased as the CDI experiment went on (Figure 6b). A higher-applied voltage could also make the desalination process faster. The falls of the rates were sharp at the beginning and the curves of the rates became much milder afterwards.

Figure 6c shows the variations in lithium-ion concentrations around the cathodes during desalination experiments. Throughout the CDI tests, the lithium-ion concentrations increased with a positive correlation between the lithium-ion concentration and the SAC value, and the release of lithium was faster when the applied voltage was higher. Before the sixth hour, the lithium-ion release curves of various voltages were tangled up, particularly for 1.0 V and 0.8 V. The final lithium-ion concentrations in the LiCl solutions were arranged in the same order as the voltages. The lowest desalination capacity of the three voltages was obtained at 0.6 V, which might be because 0.6 V did not approach the voltage plateau of spinel LMO (Figure 5b), and it was also apparent that the lithium release was negligibly little when applied at this voltage.

Desalination tests to investigate the influence of the initial NaCl solution concentration on the CDI performance were also conducted. As shown in Figure 6d, the higher concentration of the salt solution enhanced the CDI performance. More salt ions could provide not only higher conductivity in the electrolyte that could facilitate the charge and ion transfer but also a higher initial reactant concentration that would accelerate the reaction. An ultrahigh desalination capacity of 159.49 mg·g^−1^ was achieved, the comparison of which with other reported performances is illustrated in Table 1.

### 3.5. Simulation

**Figure 7:**
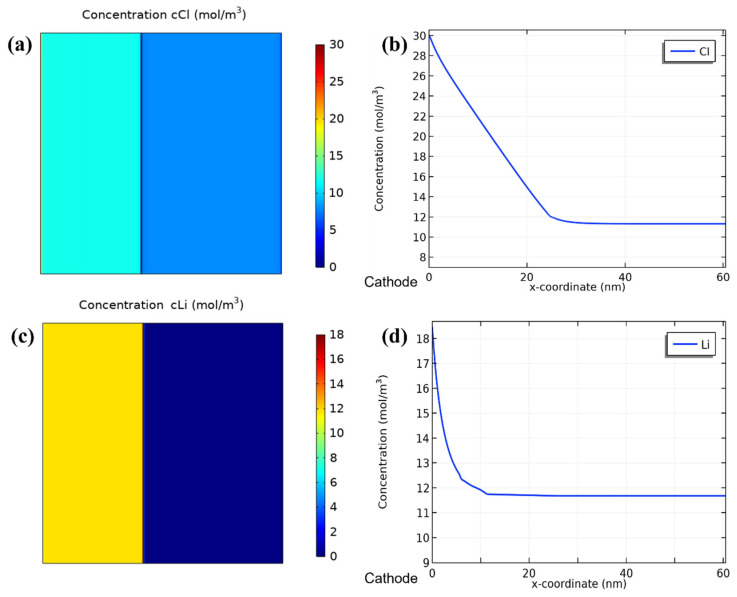
Simulated concentration distributions of chloride ions (**a**) in the device and (**b**) near the cathode after 20 h of deionization with an applied voltage of 1.0 V and an initial NaCl concentration of 10 mM; (**c**,**d**) the corresponding simulated concentration distributions of lithium ions.

The electric field and ion concentration distributions were simulated in the 2-electrode system and the results were obtained in the form of 2-dimensional plots. The initial salinity was set to be 10 mM. There were no solutions of stable state in the simulation because of the consistently changing concentrations in the device, owing to the electric field and ion exchange membrane. Thus, we chose 1 h as the iterative step to calculate the ion distributions after 20 h of desalination as shown in Figure 7. According to the calculation result, the concentrations of the ions were homogeneous in the bulk regions of the solutions while there could be some concentration changes on the solution boundary regions. In Figure 7b,d, chloride ions and lithium ions are adsorbed on the cathode and anion exchange membrane, respectively. The concentrations declined continuously with the expansion of distances between ions and the boundaries until the concentrations reached equilibrium. The thicknesses of the electrical double layers near the cathode and on the cathodic side of the membrane were both calculated to be 25.88 nm. After the deionization simulation at 1.0 V, the chloride ion concentration rose to 11.67 mM in the LiCl solution and decreased to 7.87 mM in the NaCl solution, suggesting the transfer of chloride ions through the membrane and good desalination ability of the system. The lithium ions were all located in the LiCl side of the device and the increase in the concentration of lithium ions signified their release from LMO. The simulated concentration distributions were consistent with the experiment results in 10 mM at 1.0 V.

**Figure 8:**
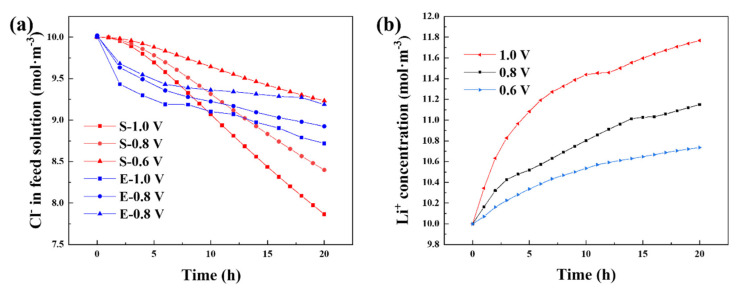
(**a**) Comparison of the simulated and experimental concentration–time curves of chloride ions in NaCl solution during the desalination processes at different voltages (red S for simulation results and blue E for experiment results); (**b**) corresponding COMSOL Multiphysics simulation results of lithium-ion concentration variations in LiCl solution.

As depicted in Figure 8a, the concentration variations of NaCl solutions in CDI processes with an initial salt concentration of 10 mM in experiment and simulation exhibited similar results and tendencies. With the same initial NaCl concentration, a higher desalination capacity could be achieved with a longer deionization time and a higher applied voltage. Compared to the simulation data, the differences between the desalination performances at different voltages were smaller and the salt removal rates were faster in the initial hours in experiments. The simulation of lithium release (Figure 8b) fitted well with the experimental profile in Figure 6c and showed the same effects from reaction time and voltage. Briefly, the simulation had confirmed the result and mechanism of the CDI desalination experiment with the spinel LMO cathode.

## 4. Conclusions

In this study, the spinel LiMn_2_O_4_ was employed as the electrode material in the HCDI cell with an anion exchange membrane, and an ultrahigh desalination capacity of 159.49 mg·g^−1^ was achieved. The deionization capacity of the system at 1.0 V with an initial salt concentration of 10 mM (91.04 mg·g^−1^) was 128.28% higher than that of the cell with anion exchange membrane incorporation and two activated carbon electrodes (39.88 mg·g^−1^), and over ten times higher than that of the cell with carbon electrodes but without membranes (7.84 mg·g^−1^). Additionally, the COMSOL Multiphysics simulation also supported the HCDI cell’s deionization capabilities and mechanism. Spinel LiMn_2_O_4_ could not only enhanced the ion transfer during deionization, but also offer hope for resolving the conflict between electrode material production and CDI performance in the desalination industry.

## Figures and Tables

**Table 1:** The comparison of CDI desalination capacities with different electrode materials.

Electrode Materials	Applied Voltage (V)	Initial Salinity	CDI Capacity (mg·g^−1^)	Reference
Na_4_Mn_9_O_18_||AC	1.2	50 mM	31.2	[14]
Na_0.71_CoO_2_||Ag/rGO	1.4	500 mg·L^−1^	31	[33]
Na_4_Ti_9_O_20_/C||AC	1.4	500 μS·cm^−1^	80.56	[34]
Na_3_V_2_(PO_4_)_3_/C||AC	1.0	100 mM	137.2	[35]
Na_2_FeP_2_O_7_/C||AC	1.2	100 mM	32.6	[36]
NiCo_2_O_4_||AC	1.2	1000 μS·cm^−1^	44.3	[37]
MoS_2_/CNT||MoS_2_/CNT	0.8	500 mM	25	[38]
Ar-modified Ti_3_C_2_T_x_||AC	1.2	500 mg·L^−1^	26.8	[39]
AC||Bi	1.2	500 mg·L^−1^	55.52	[16]
mesoporous carbon||Ag	1.2	1 mM	20.82	[40]
spinel LiMn_2_O_4_||AC	1.0	20 mM	159.49	This study

## Data Availability

Data available on request due to restrictions eg privacy or ethical. The data presented in this study are available on request from the corresponding author. The data are not publicly available due to the requests of the research group.

## Supplementary Materials

The following supporting information can be downloaded at: https://www.mdpi.com/article/10.3390/ijerph20010517/s1 [41,42].
