# Supplementary material for: Spinel LiMn2O4 as a Capacitive Deionization Electrode Material with High Desalination Capacity: Experiment and Simulation
Source: Int J Environ Res Public Health. 2022 Dec 28;20(1):517. doi: 10.3390/ijerph20010517 (PMC9819693; doi:10.3390/ijerph20010517)
Supplement: Supplementary file 1 [file ijerph-20-00517-s001.zip › ijerph-2127537-supplementary.pdf]

Supporting Information

Spinel $LiMn_2O_4$ as a Capacitive Deionization Electrode Material with High Desalination Capacity: Experiment and Simulation

Yuxin Jiang [1], Ken Li [1], Sikpaam Issaka Alhassan [2], Yiyun Cao [1], Haoyu Deng [1], Shan Tan [1], Haiying Wang [1,3,4,*], Chongjian Tang [1,3,4,*], and Liyuan Chai [1,3,4]

[1] School of Metallurgy and Environment, Central South University, Changsha 410083, China

[2] College of Engineering, Chemical and Environmental Engineering Department, University of Arizona, Tucson, AZ 85721, USA

[3] Chinese National Engineering Center for Control and Treatment of Heavy Metal Pollution, Changsha 410083, China

[4] Water Pollution Control Technology Key Lab of Hunan Province, Changsha 410083, China

* Corresponding authors.

Email addresses: haiyw25@csu.edu.cn (Haiying Wang)

chjtang@csu.edu.cn (Chongjian Tang)

1. Simulation

The controlling equations are the mathematical foundation of the simulation. The mass balance of dilute matter in the solution could be depicted by Eq. S1:

$$\frac{\partial i}{\partial t} + \nabla \cdot \boldsymbol{N_i} = R_{i,tot} \qquad \textbf{(S1)}$$

in which c_i represents of concentration of the ion i, *t* denotes the reaction time, N_i is the total flux of the matter i. The flux in the electrolyte was calculated with Nernst-Planck Equation Eq. S2:

$$\boldsymbol{N_i} = -D_i \nabla_i - Z_i u_{m,i} F c_i \nabla \emptyset_i + c_i \boldsymbol{u} = \boldsymbol{J_i} + c_i \boldsymbol{u} \qquad \textbf{(S2)}$$

where the first, second, and third terms on the left side refer to diffusion, immigration and convection, c_i represents of concentration of the ion i, Z_i is the valence of the ion, D_i refers to the diffusion coefficient, $u_{m,i}$ denotes the transfer rate of the ion, F demonstrates the Faraday constant (96485 $C \cdot mol^{-1}$), $\emptyset_i$ stands for the potential of the solution, u represents the velocity vector and $\boldsymbol{J_i}$ denotes the mole flux of relative transfer. The calculation of $\boldsymbol{J_i}$ is in the following equation:

$$\boldsymbol{J_i} = -D_i \nabla_i - Z_i u_{m,i} F c_i \nabla \emptyset_i \qquad \textbf{(S3)}$$

Electrostatic current density could be calculated by summing the fluxes:

$$i_l = F \sum Z_i \boldsymbol{N_i} + F\boldsymbol{J_H} - F\boldsymbol{J_{OH}} \qquad \textbf{(S4)}$$

in which i_l is the current density vector in the electrolyte, $\boldsymbol{J_H}$ and $\boldsymbol{J_{OH}}$ denote the mole fluxes of H^+ and OH^-, respectively. Since the electrolysis of water was introduced, the mole fluxes of H^+ and OH^- were calculated with the equations below:

$$\boldsymbol{J_H} = -D_H \nabla_{C_H} - u_{m,H} F c_H \nabla \emptyset_i \qquad \textbf{(S5)}$$

$$\boldsymbol{J_{OH}} = -D_{OH} \nabla_{C_{OH}} - u_{m,OH} F c_{OH} \nabla \emptyset_i \qquad \textbf{(S6)}$$

where c_H represents of concentration of H^+, c_{OH} represents of concentration of OH^-. The electric neutrality of the solution was controlled by Eq. S7:

$$\sum Z_i c_i + C_H - C_{OH} = 0 \qquad \textbf{(S7)}$$

To enhance the accuracy of the calculation, Eq. S2 was combined with the Poisson Equation Eq. (S8):

$$\nabla^2 \varphi = f \qquad \textbf{(S8)}$$

in which φ and f are real or complex-valued functions on a manifold. There were

no more other matters in the experiment, so we set all the boundaries in the model (except for both sides of the membrane) as no-flux boundary conditions with the following controlling equation:

$$-n \cdot J_i = 0 \tag{S9}$$

where n is normal vector of the boundary pointing into the domain. Furthermore, all the boundaries except the cathode and anode plates were set to be insulated as shown in Eq. S10:

$$-n \cdot i_l = 0 \qquad -n \cdot i_s = 0 \tag{S10}$$

in which i_l and i_s denote the current density vectors of electrolyte and electrode, respectively. There was positive charge on the ion exchange membrane and the mass/volume fraction of the electrolyte was 0.6. There is no convection because the solutions are static. The parameters of the model are presented in Table S1, where the exchange current densities of the electrodes were measured with the electrochemical workstation (Multi autolab/M204, Metrohm, Switzerland).

Table S1 Parameters of the simulation model

Symbol	Significance	Value	Reference
T	experimental temperature	298.15 K	assumed
D-Li^+	diffusion coefficient of Li^+	1.334×10^{-9} $m^2\cdot s^{-1}$	(Seader et al., 1998)
D-Cl^-	diffusion coefficient of Cl^-	2.031×10^{-9} $m^2\cdot s^{-1}$	(Seader et al., 1998)
D-Na^+	diffusion coefficient of Na^+	1.030×10^{-9} $m^2\cdot s^{-1}$	(Seader et al., 1998)
i_0-C	exchange current density of the anode	0.004486 $A\cdot m^{-2}$	measured
i_0-LMO	exchange current density of the cathode	0.002258 $A\cdot m^{-2}$	measured
α_a	anodic transfer coefficient of faradaic reaction	0.5	(Wu et al., 2014)
α_c	cathodic transfer coefficient of faradaic reaction	0.5	(Wu et al., 2014)

2. Deionization test

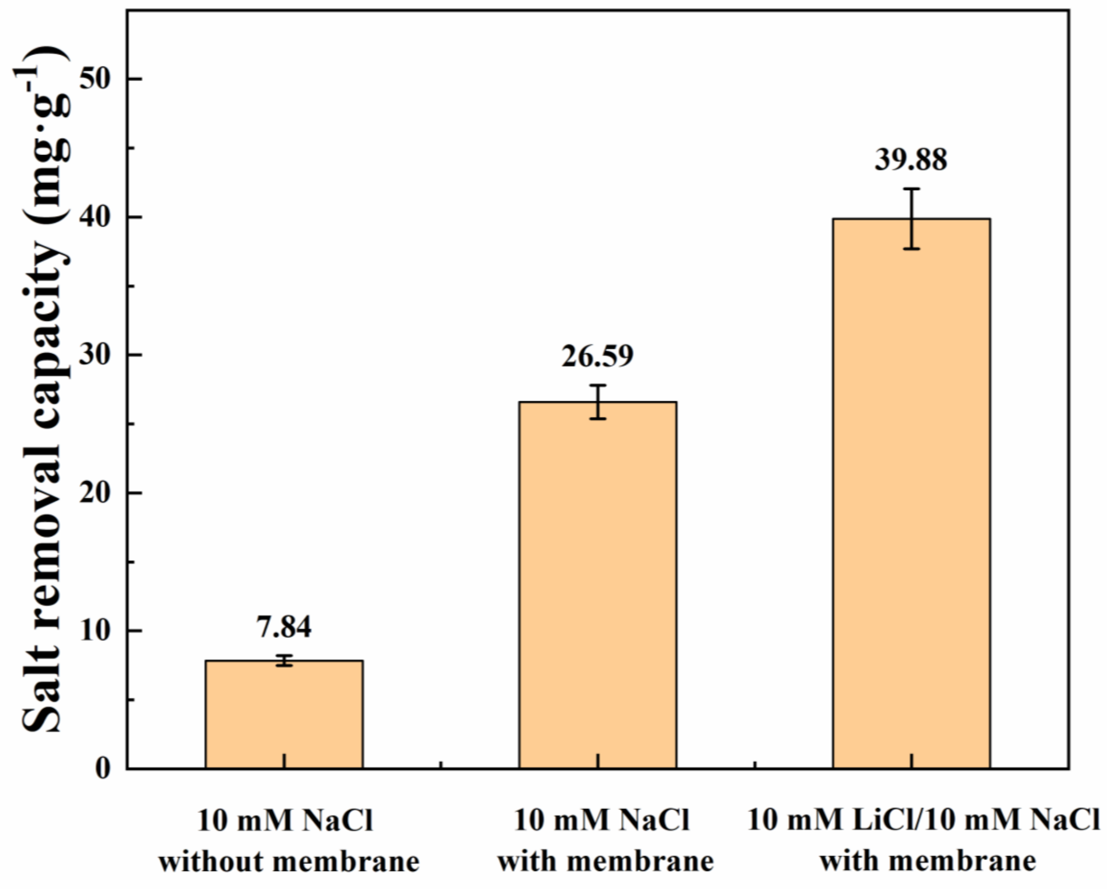

Figure S1. Comparison of the desalination capacities of different CDI cells containing only activated carbon electrodes.

3. Characterization

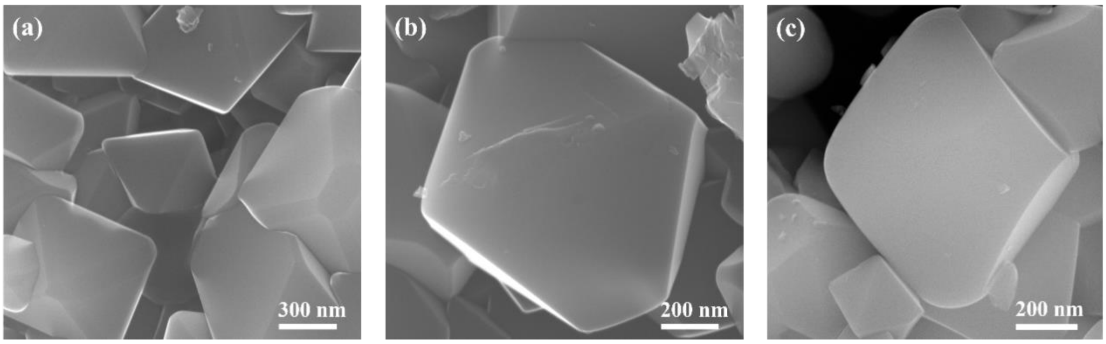

Figure S2. FESEM images of spinel $LiMn_2O_4$.

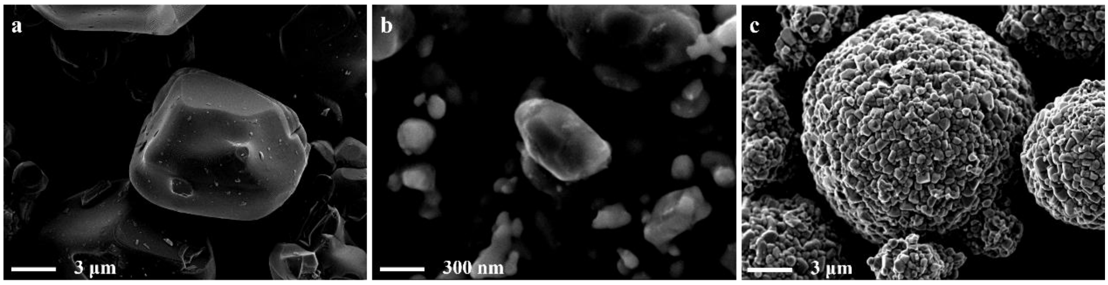

Figure S3. Field emission scanning electron microscope images of (a) $LiCoO_2$, (b) $LiFePO_4$, and (c) $LiNi_{0.5}Mn_{0.3}Co_{0.2}O_2$.

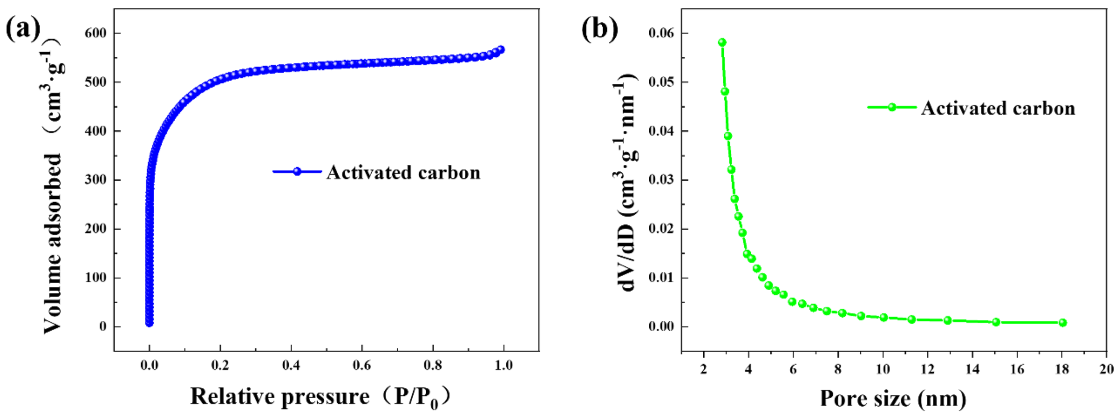

Figure S4. (a) N_2 adsorption/desorption curve and (b) pore size distribution of the activated carbon.

References

Seader, J D, Henley, E J, Roper, D K, 1998. Separation process principles. wiley New York.

Wu, W, Wang, L, Li, Y, Zhang, F, Lin, L, Niu, S, Chenet, D, Zhang, X, Hao, Y, Heinz, T F, 2014. Piezoelectricity of single-atomic-layer MoS_2 for energy conversion and piezotronics. Nature 514, 470-474.
